# Public beliefs about treatment and outcome of mental disorders: a comparison of Australia and Japan

**DOI:** 10.1186/1741-7015-3-12

**Published:** 2005-07-09

**Authors:** Anthony F Jorm, Yoshibumi Nakane, Helen Christensen, Kumiko Yoshioka, Kathleen M Griffiths, Yuji Wata

**Affiliations:** 1 ORYGEN Research Centre, Department of Psychiatry, University of Melbourne, Locked Bag 10, Parkville, Victoria 3052, Australia; 2 Centre for Mental Health Research, Australian National University, Canberra, ACT 0200, Australia; 3 Department of Social Work, The Faculty of Human Sociology, Nagasaki International University, 2825-7 Huis Ten Bosch-cho, Sasebo-shi, Nagasaki, 859-3298, Japan; 4 Department of Human Studies, Bunkyo Gakuin University,1196 Kamekubo, Oi-machi, Iruma-gun, Saitama 356-8533, Japan

## Abstract

**Background:**

Surveys of the public in a number of countries have shown poor recognition of mental disorders and beliefs about treatment that often diverge from those of health professionals. This lack of mental health literacy can limit the optimal use of treatment services. Australia and Japan are countries with very different mental health care systems, with Japan emphasising hospital care and Australia more oriented to community care. Japan is also more collectivist and Australia more individualist in values. These differences might influence recognition of disorders and beliefs about treatment in the two countries.

**Methods:**

Surveys of the public were carried out in each country using as similar a methodology as feasible. In both countries, household interviews were carried out concerning beliefs in relation to one of four case vignettes, describing either depression, depression with suicidal thoughts, early schizophrenia or chronic schizophrenia. In Australia, the survey involved a national sample of 3998 adults aged 18 years or over. In Japan, the survey involved 2000 adults aged between 20 and 69 from 25 regional sites spread across the country.

**Results:**

The Japanese public were found to be more reluctant to use psychiatric labels, particularly for the depression cases. The Japanese were also more reluctant to discuss mental disorders with others outside the family. They had a strong belief in counsellors, but not in GPs. They generally believe in the benefits of treatment, but are not optimistic about full recovery. By contrast, Australians used psychiatric labels more often, particularly "depression". They were also more positive about the benefits of seeking professional help, but had a strong preference for lifestyle interventions and tended to be negative about some psychiatric medications. Australians were positive about both counsellors and GPs. Psychiatric hospitalization and ECT were seen negatively in both countries.

**Conclusion:**

There are some major differences between Australia and Japan in recognition of disorders and beliefs about treatment. Some of these may relate to the different health care systems, but the increasing openness about mental health in Australia is also likely to be an explanatory factor.

## Background

While there is now a range of effective methods for the treatment and management of various mental disorders, many people still receive no professional help or do not receive optimal help [1]. There are many factors that affect this unmet need for treatment. One of these is a lack of mental health literacy on the part of the public, specifically a lack of knowledge of how to recognise mental disorders and beliefs about treatment that are at variance with those of health professionals [2].

Surveys in several countries have found that many members of the public do not correctly recognise disorders in a case vignette [3-5] and that they misunderstand terms such as "schizophrenia" and "mania" [6-8]. Failure to use correct psychiatric labels may cause problems of communication with health practitioners. For example, it is known that GPs are more likely to detect a mental disorder if the patient presents the symptoms in psychological rather than somatic terms [9,10], and if the patient explicitly raises the possibility of a mental disorder with the GP [11,12].

Beliefs about various types of professional help are also important. For example, if a person with a mental disorder believes that consulting a psychiatrist or psychologist is unlikely to be helpful, this will reduce their chance of getting appropriate help. Surveys in Australia and Germany have found that psychiatrists and psychologists are rated less highly than GPs for depression, but are more likely to be seen as helpful for schizophrenia [8,13].

Beliefs about types of treatment also play a role. Surveys in several countries have found predominantly negative attitudes towards psychotropic medication [7,13-18], both because of concern about side effects and the belief that medications only deal with the symptoms rather than the causes [15-18]. Such beliefs may affect adherence to prescribed medication. By contrast, psychological therapies are seen more positively [7,13,16,18-20], as are complementary therapies such as vitamins and herbs [13,20].

While surveys of public beliefs have been carried out in a number of countries, little is known about cross-cultural differences in mental health literacy. In the present paper we report data from surveys in Australia and Japan that were carried out at the same time using as similar a methodology as possible. The contrast between these two countries is interesting because of their very different systems of mental health care. While both countries have a high level of economic development and a high standard of health care, Australia places more emphasis on community care of people with mental disorders and more attention is paid to the high prevalence disorders such as depression. By contrast, in Japan there is more emphasis on hospital care, with much longer in-patient stays than in Australia, and the mental health system is largely concerned with the lower prevalence psychotic disorders. The factors leading to the emphasis on hospital care in Japan include: financial incentives for private hospital in-patient treatment under the national insurance scheme, a lack of community support programs, and the strong stigma against people with mental disorders [21]. Australia also has a system reliant on general practitioners as the first point of call for any health problem and as the gatekeepers to specialist care. By contrast, in Japan there is no specific training in primary care. Family practitioners with offices in the community provide primary care, but they are trained in other specialities [21]. Another difference between the countries, supported by informal observations, is that psychological treatments are more prominent in Australia than in Japan. There may also be cultural differences in the acceptability of expressing negative emotions and displaying behavior that departs from social norms, with Australian society being more individualist and Japanese society more collectivist. Given these differences, we expected some major differences in knowledge and beliefs about mental disorders and their treatment between the two countries.

## Methods

### Survey interview

Interview questionnaires were developed for each country to have a common core of questions that would allow comparisons between countries, and a country-specific component to allow investigation of issues particular to each country. The common core was based on the interview used in an earlier Australian survey [2], but with additional questions. The interview was based on a vignette of a person with a mental disorder. On a random basis, respondents were shown one of four vignettes: a person with major depression, one with major depression together with suicidal thoughts, a person with early schizophrenia, and one with chronic schizophrenia. All vignettes were written to satisfy the diagnostic criteria for either major depression or schizophrenia according to DSM-IV and ICD-10. The vignette with depression and the one with early schizophrenia were written to satisfy at a minimal level these diagnostic criteria, so that we could ascertain the public's reaction to cases of developing disorder that had reached the point where intervention was needed. The vignette of the person with depression together with suicidal thoughts was identical to the depression vignette in all respects except the suicidal thoughts and was designed to assess how this symptom affected the public's response. The chronic schizophrenia vignette was designed to assess the response to someone with a severe long-standing disorder, where acceptance seemed less likely. Respondents were also randomly assigned to receive either male ("John") or female ("Mary") versions of the vignette.

The depression vignette (John version) was:

John is 30 years old. He has been feeling unusually sad and miserable for the last few weeks. Even though he is tired all the time, he has trouble sleeping nearly every night. John doesn't feel like eating and has lost weight. He can't keep his mind on his work and puts off making decisions. Even day-to-day tasks seem too much for him. This has come to the attention of his boss, who is concerned about John's lowered productivity.

The depression with suicidal thoughts vignette was:

John is 30 years old. He has been feeling unusually sad and miserable for the last few weeks. Even though he is tired all the time, he has trouble sleeping nearly every night. John doesn't feel like eating and has lost weight. He can't keep his mind on his work and puts off making any decisions. Even day-to-day tasks seem too much for him. This has come to the attention of John's boss who is concerned about his lowered productivity. John feels he will never be happy again and believes his family would be better off without him. John has been so desperate, he has been thinking of ways to end his life.

The early schizophrenia vignette was:

John is 24 and lives at home with his parents. He has had a few temporary jobs since finishing school but is now unemployed. Over the last six months he has stopped seeing his friends and has begun locking himself in his bedroom and refusing to eat with the family or to have a bath. His parents also hear him walking about his bedroom at night while they are in bed. Even though they know he is alone, they have heard him shouting and arguing as if someone else is there. When they try to encourage him to do more things, he whispers that he won't leave home because he is being spied upon by the neighbour. They realize he is not taking drugs because he never sees anyone or goes anywhere.

The chronic schizophrenia vignette was:

John is 44 years old. He is living in a boarding house in an industrial area. He has not worked for years. He wears the same clothes in all weathers and has left his hair to grow long and untidy. He is always on his own and is often seen sitting in the park talking to himself. At times he stands and moves his hands as if to communicate to someone in nearby trees. He rarely drinks alcohol. He speaks carefully using uncommon and sometimes made-up words. He is polite but avoids talking with other people. At times he accuses shopkeepers of giving information about him to other people. He has asked his landlord to put extra locks on his door and to remove the television set from his room. He says spies are trying to keep him under observation because he has secret information about international computer systems which control people through television transmitters. His landlord complains that he will not let him clean the room which is increasingly dirty and filled with glass objects. John says he is using these "to receive messages from space".

After being presented with the vignette, respondents were asked two open-ended questions: "What would you say, if anything, is wrong with John/Mary?" and "How do you think John/Mary could best be helped?" Then followed a series of questions asking the respondent to rate the likely helpfulness of various interventions (rated as likely to be helpful, harmful or neither for the person in the vignette). The interventions were: a typical GP or family doctor; a typical chemist (pharmacist); a counselor; a social worker; a telephone counseling service, such as Lifeline; a psychiatrist; a psychologist; help from close family; help from close friends; a naturopath or a herbalist; the clergy, a minister or priest; John/Mary tried to deal with his/her problems on his/her own; vitamins and mineral, tonics or herbal medicines; pain relievers, such as aspirin, codeine or panadol; antidepressants; antibiotics; sleeping pills; anti-psychotics; tranquillizers such as valium; becoming physically more active, such as playing more sport, or doing a lot more walking or gardening; reading about people with similar problems and how they have dealt with them; getting out and about more; attending courses or relaxation, stress management, meditation or yoga; cutting out alcohol altogether; psychotherapy; hypnosis; being admitted to a psychiatric ward of a hospital; undergoing electro-convulsive therapy (ECT); having an occasional alcoholic drink to relax; going on a special diet or avoiding certain foods. Next were questions asking about the likely result for the person in the vignette with and without "the sort of professional help you think is most appropriate" The response options were: Full recovery with no further problems; Full recovery, but problems would probably re-occur; Partial recovery; Partial recovery, but problems would probably re-occur; No improvement; Get worse.

The rest of the common core interview is not relevant to the analyses reported here; it involved questions on knowledge of causes and risk factors, beliefs associated with stigma and discrimination, contact with people like those in the vignette, and the health of the respondent.

### The Australian survey

A household survey was carried out of Australian adults aged 18 or over by the company AC Nielsen. Households were sampled from 250 census districts covering all states and territories and metropolitan and rural areas. Up to 5 call backs were made to metropolitan selections and 3 to non-metropolitan selections. Interviews were sought with the person in the household who had the most recent birthday. To achieve a target sample of 4,000 interviews with adults aged 18 years or over, visits were made to 28,947 households. The outcome of these visits was: no contact after repeated visits 14,630; vacant house or lot 306; refused 7,815; person sampled within household temporarily unavailable 1,132; no suitable respondent in household 287; did not speak English 383; incapable of responding 213; and unavailable for the duration of the survey 181. The achieved sample was 3998 persons, with 1001 receiving the depression vignette, 999 the depression with suicidal thoughts vignette, 997 the early schizophrenia vignette, and 1001 the chronic schizophrenia vignette.

In addition to the common core component, the Australian survey interview had questions about awareness of depression in the media and about Australia's national depression initiative.

Ethics approval was given by the Human Research Ethics Committee of the Australian National University.

### The Japanese survey

A survey manual supplied from Australia was translated into Japanese and entrusted to Yamate Information Processing Center Ltd. for use with the target population aged 20–69 years, as a rule using the same procedures as Australia. The survey questionnaire, which was developed by the Australian researchers (AFJ, HC, KMG), was tentatively translated into Japanese. Then a native English translator, who had not seen the original English text, translated the Japanese version back into English. By comparing the two English versions, it was possible to confirm the accuracy of the original translation. There were no significant differences between the original text and the reverse translation. Finally, a Japanese version of the questionnaire was produced, which involved formatting the text into Japanese style and making slight wording adjustments. The names of the characters in the vignettes were translated into the Japanese style, viz. "A-o"(putting an o sound at the end is often used for a man's name) or "B-ko" (putting ko at the end is often used for a woman's name), instead of "John" or "Mary" which were used in the English text.

As well as the questions taken from the Australian survey, the Japanese survey asked questions concerning such issues as psychiatric health and welfare policy, the bodies implementing related services, the existence of action groups, and the change in the Japanese name for schizophrenia by the Japanese Society of Psychiatry and Neurology (i.e. from "split personality disorder" to "schizophrenia"). These additions were made to clarify the current Japanese situation and issues in related fields. Further, an original Japanese manual was also created and adopted for use concerning points of interest in the implementation of home visits.

The survey method used was home visit interviews. It was not feasible to do a national survey of randomly selected households in Japan because of constraints of human resources, funding and time. It was therefore decided to sample a range of areas that differed in whether they were large or small cities, whether the area had many psychiatric patients or not, and whether the area had a high suicide rate or not. Using this approach, Japan was divided into 5 areas and 5 research sites were selected in each of these areas, giving a total of 25 geographic sites. As the survey was conducted during the winter, and because it was difficult to ensure that there would be enough survey interviewers, implementation in Hokkaido and Shikoku prefectures proved troublesome. Additional reasons for selection of the 25 regional sites were that they were places of comparatively high population within the relevant regions, the survey interviewers could use public transport, and the urban areas involved no particular inconveniences for the researchers to visit within a certain range using public transportation. 80 households were selected from each site, giving a total of 2000. At each site there were 4 interviewers who took responsibility for 20 households each. The survey was conducted over the period from 19 November to 12 December 2003. Each of the four vignettes was received by 250 people. Half received a male version of a vignette and half the female version.

At the start of the survey, an explanatory meeting was held for the survey interviewers in each region. As many members of the research team as possible attended these explanatory meetings. Eighty-five survey interviewers were recruited for this research with an average age of 50 and an average of 17 years' experience of interviewing in various types of surveys. The areas for the survey interviewers to canvass were allocated on the basis of where they lived. The question of where the individual survey interviewers should go was determined mutually among the survey interviewers themselves, and by the head survey interviewer (supervisor). As a rule, one survey interviewer conducted 20 interviews, but this was considerably flexible, given the number of years of individual experience and what the individual survey interviewer could handle. The interviews were conducted according to the following procedure: visit the target's home and present the written greetings and request (a draft had been prepared by certain survey bodies, which was put into final form after checks by the research team members), then explain the details of the survey using the documents, ask the target for their participation in the research, start the interview and follow through to completion, check that nothing had been omitted from the survey responses, and hand over the remuneration (1000 yen cash voucher). Data were not collected on the refusal rate for this survey.

### Statistical analysis

Data were pooled across male and female versions of each vignette and percent frequencies calculated. For the Australian survey, percentages were calculated applying survey weights to give better population estimates. Ninety-five percent CIs were estimated using the Complex Samples procedure in SPSS 12.0. This procedure takes account of sampling weights and geographic clustering in the sample. For the Japanese survey, percentage frequencies and 95% CIs were calculated using unweighted data with SPSS 12.0.

Because of the very different cultures of Australia and Japan, it is possible that any differences in question endorsement rates might be due to subtleties of language or to the social rules applying to the interview situation, as well as to genuine differences in beliefs about treatment and outcome. For this reason, we have not relied on statistical significance of absolute percentages between countries, but rather on the broad patterns of responses, particularly where percent endorsement was ordered very differently across questions.

## Results

### Recognition of disorders

Table 1 shows the results from both countries. In Australia, "depression" was the term used most often to describe both the depression vignette and the depression with suicidal thoughts vignette. "Schizophrenia/psychosis" was the term used most often to describe both of the schizophrenia vignettes, while the generic term "mental illness" was also commonly used for these vignettes.

**Table 1:** Percentage (and 95% CI) of respondents mentioning each category to describe the problem shown in the vignette

**Category mentioned**	**Country**	**Depression Vignette**	**Depression/Suicidal Vignette**	**Early Schizophrenia Vignette**	**Chronic Schizophrenia Vignette**
Depression	Australia	65.3 (60.5–69.8)	77.3 (72.7–81.3)	34.8 (30.5–39.4)	9.6 (7.0–13.0)
	Japan	22.6 (18.9–26.3)	35.0 (30.8–39.2)	13.6 (10.6–16.6)	9.6 (7.0–12.2)
Schizophrenia/Psychosis	Australia	0.0 (0.0–0.0)	0.5 (0.1–1.6)	41.2 (36.5–46.0)	36.1 (31.5–40.9)
	Japan	2.2 (0.9–3.5)	1.2 (0.2–2.2)	17.2 (13.9–20.5)	33.4 (29.3–37.5)
Nervous breakdown	Australia	0.7 (0.3–2.1)	1.6 (0.8–3.3)	1.7 (0.9–3.2)	1.0 (0.3–3.4)
	Japan	2.0 (0.8–3.2)	2.6 (1.2–4.0)	2.6 (1.2–4.0)	2.4 (1.1–3.7)
Psychological/Mental/Emotional problems	Australia	4.5 (2.9–6.8)	6.0 (4.2–8.7)	12.9 (10.1–16.3)	14.3 (10.9–18.5)
	Japan	29.4 (25.4–33.4)	24.8 (21.0–28.6)	28.4 (24.4–32.4)	27.2 (23.3–31.1)
Mental illness	Australia	3.0 (1.7–5.1)	5.5 (3.7–8.2)	23.0 (19.4–27.0)	35.8 (31.4–40.4)
	Japan	9.2 (6.7–11.7)	10.2 (7.5–12.9)	21.6 (18.0–25.2)	12.8 (9.9–15.7)
Stress	Australia	16.6 (13.1–20.8)	10.9 (8.3–14.3)	3.1 (1.8–5.3)	2.8 (1.4–5.5)
	Japan	25.0 (21.2–28.8)	19.8 (16.3–23.3)	5.0 (3.1–6.9)	3.8 (2.1–5.5)

In Japan, no single term predominated for describing the depression vignettes, with "depression", "stress" and "psychological/mental/emotional problems" being the most common. For the early schizophrenia vignette, the generic categories of "mental illness" and "psychological/mental/emotional problems" were used most frequently, while for the chronic schizophrenia vignette, "schizophrenia" and "psychological/mental/emotional problems" were most commonly used.

### Best method of help

Table 2 shows the frequency of various responses to the open-ended question about how the person in the vignette could best be helped. In Australia, half the respondents mentioned seeing a GP for the depression vignettes. Other common responses to the depression vignettes were seeing a counselor or talking with friends or family. For the schizophrenia vignettes, seeing a psychiatrist was commonly mentioned, in addition to seeing a GP or counselor or talking with friends or family.

**Table 2:** Percentage (and 95% CI) of respondents mentioning each category in response to the open-ended question about how the person in the vignette could best be helped

**Type of help mentioned**	**Country**	**Depression Vignette**	**Depression/Suicidal Vignette**	**Early Schizophrenia Vignette**	**Chronic Schizophrenia Vignette**
See a doctor/GP	Australia	56.3 (52.5–60.0)	49.3 (44.4–54.1)	32.1 (27.6–37.0)	21.0 (17.2–25.4)
	Japan	20.8 (17.2–24.4)	20.0 (16.5–23.5)	13.4 (10.4–16.4)	15.8 (12.6–19.0)
See a psychiatrist	Australia	13.0 (10.7–15.6)	18.2 (14.9–22.0)	32.0 (27.8–36.4)	27.9 (23.6–32.6)
	Japan	43.8 (39.4–48.2)	49.0 (44.6–53.4)	58.6 (54.3–62.9)	60.0 (55.7–64.3)
Take medication	Australia	6.1 (4.7–7.8)	8.7 (6.5–11.5)	8.3 (6.0–11.4)	11.7 (9.0–15.1)
	Japan	3.4 (1.8–5.0)	8.0 (5.6–10.4)	7.8 (5.4–10.2)	5.8 (3.7–7.9)
See a counselor or have counseling	Australia	27.7 (24.7–30.9)	37.4 (33.2–41.8)	28.9 (24.7–33.4)	20.8 (17.3–24.7)
	Japan	62.0 (57.7–66.3)	74.8 (71.0–78.6)	76.4 (72.7–80.1)	72.2 (68.3–76.1)
Talk over with friends/family	Australia	22.9 (19.8–26.4)	24.0 (20.0–28.6)	21.9 (18.0–26.3)	14.4 (11.2–18.4)
	Japan	71.8 (67.8–75.8)	70.4 (66.4–74.4)	43.8 (39.4–48.2)	45.2 (40.8–49.6)
Person must first recognize problem	Australia	5.4 (3.8–7.6)	6.6 (4.3–9.9)	5.3 (3.3–8.4)	6.0 (4.1–8.7)
	Japan	23.4 (19.7–27.1)	24.4 (20.6–28.2)	23.4 (19.7–27.1)	21.8 (18.2–25.4)
Other	Australia	37.9 (34.0–41.8)	36.0 (31.1–41.1)	40.1 (34.9–45.4)	49.8 (44.6–55.0)
	Japan	8.4 (6.0–10.8)	2.6 (1.2–4.0)	4.4 (2.6–6.2)	7.2 (4.9–9.5)
Don't know	Australia	1.8 (1.1–3.0)	2.5 (1.4–4.6)	2.0 (1.0–3.7)	4.8 (3.1–7.5)
	Japan	0.4 (0.0–1.0)	1.0 (0.1–1.9)	1.6 (0.5–2.7)	1.2 (0.2–2.2)

Note: because multiple responses were possible, these percentages do not add up to 100%

In Japan, the most commonly mentioned help for the depression vignettes was counseling and family or friends. For the schizophrenia vignettes, seeing a counselor or a psychiatrist were commonly mentioned, but talking it over with family or friends was less commonly mentioned than for the depression vignettes. Seeing a GP was seldom mentioned for any vignette.

### Beliefs about specific interventions

Tables 3, 4, 5 show the data on the ratings of likely helpfulness of interventions. The Australian public gave similar ratings for the two depression vignettes. The interventions most commonly endorsed as likely to be helpful were seeing a GP, counselor, close friends, physical activity, reading about the problem, getting out more, learning relaxation, or getting information from a health educator. The responses were similar for the schizophrenia vignettes, except that GPs were rated somewhat lower and psychiatrists somewhat higher. Seeing a social worker was also commonly endorsed for the chronic schizophrenia vignette.

**Table 3:** Percentage (95% CI) of respondents rating each type of person as "helpful" for the person described in the vignette

**Person**	**Country**	**Depression Vignette**	**Depression/Suicidal Vignette**	**Early Schizophrenia Vignette**	**Chronic Schizophrenia Vignette**
GP	Australia	87.3 (84.0–90.0)	84.1 (80.8–87.0)	76.7 (72.8–80.2)	76.3 (72.1–80.0)
	Japan	30.4 (26.4–34.4)	26.0 (22.1–29.9)	19.0 (15.5–22.5)	22.8 (19.1–26.5)
Pharmacist	Australia	35.4 (31.3–39.6)	33.2 (29.1–37.6)	23.6 (20.0–27.6)	28.1 (24.1–32.5)
	Japan	6.8 (4.6–9.0)	6.6 (4.4–8.8)	4.2 (2.4–6.0)	4.2 (2.4–6.0)
Counselor	Australia	82.2 (78.6–85.4)	85.5 (82.1–88.3)	85.0 (81.8–87.8)	83.1 (79.5–86.1)
	Japan	85.8 (82.7–88.9)	87.6 (84.7–90.5)	87.0 (84.0–90.0)	88.6 (85.8–91.4)
Social worker	Australia	62.8 (58.3–67.0)	67.2 (62.8–71.4)	68.4 (64.0–72.5)	79.1 (74.8–82.8)
	Japan	73.4 (69.5–77.3)	70.2 (66.2–74.2)	68.4 (64.3–72.5)	75.2 (71.4–79.0)
Phone counseling	Australia	63.5 (59.0–67.9)	66.2 (61.8–70.4)	56.6 (52.4–60.7)	47.5 (43.0–52.1)
	Japan	42.4 (38.1–46.7)	49.8 (45.4–54.2)	35.6 (31.4–39.8)	29.6 (25.6–33.6)
Psychiatrist	Australia	65.0 (60.8–69.0)	71.3 (67.1–75.1)	80.5 (76.5–84.0)	80.2 (76.4–83.5)
	Japan	69.4 (65.3–73.5)	72.4 (68.5–76.3)	73.0 (69.1–76.9)	79.0 (75.4–82.6)
Psychologist	Australia	66.9 (62.5–71.1)	69.7 (65.2–73.8)	73.6 (69.4–77.4)	74.9 (70.8–78.6)
	Japan	56.6 (52.2–61.0)	51.2 (46.8–55.6)	56.2 (51.8–60.6)	65.2 (61.0–69.4)
Close family	Australia	67.9 (63.1–72.3)	64.8 (60.1–69.2)	62.7 (58.4–66.8)	61.4 (56.2–66.3)
	Japan	85.0 (81.9–88.1)	84.2 (81.0–87.4)	76.8 (73.1–80.5)	80.4 (76.9–83.9)
Close friends	Australia	78.2 (74.1–81.7)	77.1 (73.2–80.5)	73.0 (68.9–76.7)	72.0 (67.5–76.1)
	Japan	84.8 (81.6–88.0)	83.2 (79.9–86.5)	70.4 (66.4–74.4)	70.2 (66.2–74.2)
Naturopath/herbalist	Australia	34.9 (30.8–39.3)	31.8 (27.5–36.5)	23.7 (20.2–27.7)	19.4 (16.3–22.9)
	Japan	11.2 (8.4–14.0)	14.8 (11.7–17.9)	8.4 (6.0–10.8)	9.0 (6.5–11.5)
Clergy	Australia	45.3 (41.0–49.7)	51.7 (47.3–56.0)	37.2 (33.1–41.4)	42.9 (38.3–47.7)
	Japan	13.6 (10.6–16.6)	20.0 (16.5–23.5)	11.6 (8.8–14.4)	16.2 (13.0–19.4)
Deal with it alone	Australia	13.1 (10.1–16.8)	9.7 (7.0–13.2)	11.4 (8.4–15.3)	11.8 (8.9–15.6)
	Japan	24.4 (20.6–28.2)	20.4 (16.9–23.9)	22.4 (18.7–26.1)	21.4 (17.8–25.0)

**Table 4:** Percentage (95% CI) of respondents rating each type of medication as "helpful" for the person described in the vignette

**Medication**	**Country**	**Depression Vignette**	**Depression/Suicidal Vignette**	**Early Schizophrenia Vignette**	**Chronic Schizophrenia Vignette**
Vitamins, minerals	Australia	50.2 (45.4–55.1)	43.7 (39.2–48.3)	31.3 (27.4–35.5)	33.2 (28.9–37.8)
	Japan	20.2 (16.7–23.7)	16.4 (13.1–19.7)	10.6 (7.9–13.3)	12.4 (9.5–15.3)
Pain relievers	Australia	14.8 (11.7–18.5)	12.3 (9.6–15.7)	7.3 (5.3–9.9)	10.2 (7.7–13.4)
	Japan	4.4 (2.6–6.2)	3.6 (2.0–5.2)	4.2 (2.4–6.0)	4.6 (2.8–6.4)
Antidepressants	Australia	46.7 (42.4–51.1)	52.5 (48.1–56.7)	49.9 (45.7–54.2)	42.6 (37.9–47.5)
	Japan	34.8 (30.6–39.0)	36.0 (31.8–40.2)	38.6 (34.3–42.9)	39.8 (35.5–44.1)
Antibiotics	Australia	10.4 (7.9–13.7)	7.9 (5.7–10.8)	4.0 (2.5–6.2)	6.4 (4.5–9.1)
	Japan	6.2 (4.1–8.3)	6.0 (3.9–8.1)	4.8 (2.9–6.7)	8.4 (6.0–10.8)
Sleeping pills	Australia	23.9 (20.1–28.1)	21.9 (18.6–25.6)	18.1 (14.7–22.1)	11.6 (8.8–15.1)
	Japan	31.6 (27.5–35.7)	26.2 (22.3–30.1)	21.4 (17.8–25.0)	24.8 (21.0–28.6)
Antipsychotics	Australia	11.2 (8.4–14.8)	16.5 (13.3–20.3)	33.1 (29.0–37.5)	38.2 (34.0–42.6)
	Japan	22.6 (18.9–26.3)	21.8 (18.2–25.4)	30.2 (26.2–34.2)	41.2 (36.9–45.5)
Tranquillizers	Australia	13.8 (11.0–17.1)	13.8 (11.0–17.1)	17.2 (14.1–20.8)	15.3 (12.6–18.4)
	Japan	38.4 (34.1–42.7)	37.0 (32.8–41.2)	38.4 (34.1–42.7)	45.4 (41.0–49.8)

**Table 5:** Percentage (95% CI) of respondents rating each type of intervention as "helpful" for the person described in the vignette

**Intervention**	**Country**	**Depression Vignette**	**Depression/Suicidal Vignette**	**Early Schizophrenia Vignette**	**Chronic Schizophrenia Vignette**
Physical activity	Australia	92.0 (89.3–94.1)	92.5 (89.9–94.4)	87.4 (84.2–90.1)	79.6 (75.6–83.1)
	Japan	69.4 (65.3–73.5)	73.4 (69.5–77.3)	73.4 (69.5–77.3)	70.6 (66.6–74.6)
Read about problem	Australia	79.3 (75.3–82.8)	79.8 (75.8–83.3)	79.6 (75.5–83.1)	74.7 (70.6–78.4)
	Japan	60.0 (55.7–64.3)	59.4 (55.1–63.7)	57.6 (53.3–61.9)	46.8 (42.4–51.2)
Get out more	Australia	87.0 (83.8–89.7)	90.3 (87.4–92.6)	87.1 (83.8–89.8)	76.5 (72.3–80.2)
	Japan	67.0 (62.9–71.1)	72.0 (68.1–75.9)	67.2 (63.1–71.3)	61.6 (57.3–65.9)
Learn relaxation	Australia	83.6 (80.1–86.7)	85.3 (81.8–88.2)	77.1 (73.3–80.5)	68.7 (64.4–72.8)
	Japan	38.2 (33.9–42.5)	41.2 (36.9–45.5)	26.2 (22.3–30.1)	29.4 (25.4–33.4)
Cut out alcohol	Australia	56.0 (51.7–60.3)	59.8 (55.3–64.1)	66.1 (61.7–70.2)	53.4 (48.7–58.0)
	Japan	10.0 (7.4–12.6)	14.2 (11.1–17.3)	18.6 (15.2–22.0)	17.2 (13.9–20.5)
Psychotherapy	Australia	44.1 (39.7–48.5)	50.4 (46.0–54.8)	59.1 (54.5–63.6)	62.3 (57.4–66.8)
	Japan	49.0 (44.6–53.4)	48.2 (43.8–52.6)	53.8 (49.4–58.2)	67.0 (62.9–71.1)
Hypnosis	Australia	22.4 (18.8–26.5)	23.9 (20.2–28.1)	29.9 (25.9–34.3)	30.9 (26.8–35.2)
	Japan	28.0 (24.1–31.9)	28.8 (24.8–32.8)	22.4 (18.7–26.1)	33.2 (29.1–37.3)
Psychiatric ward	Australia	16.4 (13.2–20.2)	20.2 (16.7–24.3)	31.9 (27.8–36.4)	37.8 (33.4–42.6)
	Japan	13.6 (10.6–16.6)	12.0 (9.1–14.9)	22.0 (18.4–25.6)	30.0 (26.0–34.0)
ECT	Australia	5.9 (4.0–8.6)	7.2 (4.9–10.4)	6.4 (4.5–9.1)	6.5 (4.4–9.4)
	Japan	2.2 (0.9–3.5)	1.4 (0.4–2.4)	1.4 (0.4–2.4)	1.4 (0.4–2.4)
Occasional drink	Australia	44.4 (40.1–48.9)	41.8 (37.1–46.5)	31.1 (26.9–34.7)	27.3 (23.1–31.9)
	Japan	31.4 (27.3–35.5)	25.0 (21.2–28.8)	15.2 (12.0–18.4)	20.0 (16.5–23.5)
Special diet	Australia	48.3 (43.5–53.1)	45.6 (41.0–50.3)	42.1 (37.9–46.3)	39.3 (35.0–43.9)
	Japan	5.6 (3.6–7.6)	6.0 (3.9–8.1)	4.4 (2.6–6.2)	4.4 (2.6–6.2)
Web site	Australia	57.9 (53.8–61.9)	55.1 (50.4–59.7)	57.5 (53.0–61.8)	44.1 (39.4–49.0)
	Japan	45.6 (41.2–50.0)	45.8 (41.4–50.2)	48.4 (44.0–52.8)	47.0 (42.6–51.4)
Expert via email	Australia	53.8 (49.6–58.0)	49.6 (44.9–54.3)	55.4 (51.2–59.5)	44.7 (40.1–49.5)
	Japan	54.0 (49.6–58.4)	53.6 (49.2–58.0)	56.8 (52.4–61.2)	56.6 (52.2–61.0)
Book	Australia	69.1 (65.3–72.6)	64.7 (60.0–69.1)	70.5 (66.5–74.2)	59.2 (54.3–63.9)
	Japan	54.0 (49.6–58.4)	49.8 (45.4–54.2)	57.4 (53.1–61.7)	53.6 (49.2–58.0)
Health educator	Australia	86.7 (83.6–89.3)	85.9 (82.3–88.8)	86.2 (83.1–88.8)	83.8 (79.7–87.2)
	Japan	55.2 (50.8–59.6)	51.2 (46.8–55.6)	46.6 (42.2–51.0)	50.6 (46.2–55.0)

In Japan, the most commonly endorsed interventions for the depression vignettes were seeing a counselor, or help from friends and family. For the schizophrenia vignettes, seeing a counselor rated highly again, as did close family. Psychiatrists and social workers were highly endorsed for the chronic schizophrenia vignette.

In neither country was there a high level of endorsement for some standard psychiatric interventions: antidepressants for the depression vignettes, antipsychotics for the schizophrenia vignettes, admission to a psychiatric ward for the schizophrenia vignettes, or psychotherapy for the depression vignettes.

Tables 6, 7, 8 show the data on whether interventions were rated as likely to be harmful. In Australia, "harmful" ratings were most common for dealing with the problem alone, sleeping pills, tranquillizers, ECT and admission to a psychiatric ward. In Japan, such ratings were most common for dealing with the problem alone, pain relievers, admission to a psychiatric ward, ECT and going on a special diet.

**Table 6:** Percentage (95% CI) of respondents rating each type of person as "harmful" for the person described in the vignette

**Person**	**Country**	**Depression Vignette**	**Depression/Suicidal Vignette**	**Early Schizophrenia Vignette**	**Chronic Schizophrenia Vignette**
GP	Australia	0.5 (0.2–1.6)	1.1 (0.5–2.5)	2.5 (1.5–4.3)	2.7 (1.5–4.7)
	Japan	9.4 (6.8–12.0)	9.0 (6.5–11.5)	12.6 (9.7–15.5)	12.8 (9.9–15.7)
Pharmacist	Australia	8.7 (6.3–12.1)	8.1 (5.7–11.5)	8.6 (6.4–11.4)	8.2 (5.8–11.5)
	Japan	23.6 (19.9–27.3)	22.0 (18.4–25.6)	22.4 (18.7–26.1)	23.0 (19.3–26.7)
Counselor	Australia	3.1 (1.8–5.3)	2.3 (1.3–3.8)	3.0 (1.8–5.0)	2.4 (1.3–4.4)
	Japan	1.0 (0.1–1.9)	1.0 (0.1–1.9)	1.4 (0.4–2.4)	1.6 (0.5–2.7)
Social worker	Australia	4.5 (3.0–6.7)	5.5 (3.6–8.2)	4.4 (3.0–6.5)	3.0 (1.8–5.0)
	Japan	1.4 (0.4–2.4)	3.0 (1.5–4.5)	4.8 (2.9–6.7)	3.0 (1.5–4.5)
Phone counseling	Australia	5.9 (4.1–8.6)	6.3 (4.2–9.2)	7.6 (5.3–10.9)	11.1 (8.4–14.6)
	Japan	8.6 (6.1–11.1)	6.6 (4.4–8.8)	11.0 (8.2–13.8)	12.0 (9.1–14.9)
Psychiatrist	Australia	7.1 (5.0–10.1)	8.1 (5.9–11.0)	5.2 (3.5–7.7)	4.6 (3.1–6.8)
	Japan	5.4 (3.4–7.4)	4.8 (2.9–6.7)	6.0 (3.9–8.1)	2.8 (1.3–4.3)
Psychologist	Australia	5.1 (3.3–7.9)	5.2 (3.5–7.7)	3.2 (2.0–5.0)	3.6 (2.3–5.6)
	Japan	6.0 (3.9–8.1)	8.0 (5.6–10.4)	6.0 (3.9–8.1)	5.0 (3.1–6.9)
Close family	Australia	4.9 (3.3–7.1)	4.1 (2.7–6.1)	5.6 (3.9–8.0)	5.3 (3.7–7.7)
	Japan	1.6 (0.5–2.7)	1.6 (0.5–2.7)	4.6 (2.8–6.4)	4.4 (2.6–6.2)
Close friends	Australia	2.1 (1.1–3.7)	2.6 (1.5–4.5)	3.0 (1.8–4.8)	3.3 (2.0–5.3)
	Japan	1.8 (0.6–3.0)	1.4 (0.4–2.4)	4.0 (2.3–5.7)	4.2 (2.4–6.0)
Naturopath/herbalist	Australia	11.1 (8.5–14.5)	13.3 (10.5–16.7)	15.1 (12.1–18.7)	15.0 (11.8–18.9)
	Japan	18.8 (15.4–22.2)	17.2 (13.9–20.5)	18.2 (14.8–21.6)	21.4 (17.8–25.0)
Clergy	Australia	8.1 (5.8–11.1)	9.3 (7.2–11.9)	11.6 (8.9–15.0)	10.3 (7.7–13.6)
	Japan	24.2 (20.4–28.0)	14.6 (11.5–17.7)	26.0 (22.1–29.9)	24.4 (20.6–28.2)
Deal with it alone	Australia	64.0 (59.6–68.3)	74.8 (70.4–78.7)	70.4 (65.9–74.5)	67.7 (62.9–72.2)
	Japan	41.4 (37.1–45.7)	42.6 (38.3–46.9)	38.8 (34.5–43.1)	40.8 (36.5–45.1)

**Table 7:** Percentage (95% CI) of respondents rating each type of medication as "harmful" for the person described in the vignette

**Medication**	**Country**	**Depression Vignette**	**Depression/Suicidal Vignette**	**Early Schizophrenia Vignette**	**Chronic Schizophrenia Vignette**
Vitamins, minerals	Australia	4.4 (2.9–6.7)	5.4 (3.7–7.9)	5.8 (4.1–8.4)	6.4 (4.5–9.0)
	Japan	14.6 (11.5–17.7)	13.8 (10.8–16.8)	14.6 (11.5–17.7)	14.8 (11.7–17.9)
Pain relievers	Australia	37.7 (33.0–42.6)	37.3 (33.1–41.7)	38.9 (34.6–43.4)	34.5 (30.0–39.3)
	Japan	43.4 (39.0–47.8)	42.6 (38.3–46.9)	36.6 (32.4–40.8)	35.6 (31.4–39.8)
Antidepressants	Australia	27.5 (23.5–31.8)	23.4 (19.8–27.4)	22.6 (19.2–26.5)	29.3 (25.2–33.8)
	Japan	18.2 (14.8–21.6)	21.2 (17.6–24.8)	15.2 (12.0–18.4)	10.6 (7.9–13.3)
Antibiotics	Australia	38.3 (33.4–43.4)	37.8 (33.0–42.8)	35.9 (31.5–40.4)	36.9 (32.4–41.7)
	Japan	29.8 (25.8–33.8)	37.6 (33.3–41.9)	29.0 (25.0–33.0)	22.8 (19.1–26.5)
Sleeping pills	Australia	49.6 (44.9–54.3)	50.3 (45.9–54.7)	53.1 (48.2–57.9)	58.8 (54.3–63.1)
	Japan	27.0 (23.1–30.9)	27.8 (23.9–31.7)	30.0 (26.0–34.0)	27.0 (23.1–30.9)
Antipsychotics	Australia	48.3 (43.5–53.1)	40.4 (35.9–45.0)	24.5 (20.6–28.8)	24.5 (20.9–28.5)
	Japan	19.0 (15.5–22.5)	23.8 (20.1–27.5)	17.4 (14.1–20.7)	8.4 (6.0–10.8)
Tranquillizers	Australia	60.4 (55.8–64.8)	60.1 (55.5–64.5)	47.5 (42.9–52.2)	55.7 (50.9–60.4)
	Japan	15.8 (12.6–19.0)	17.6 (14.3–20.9)	13.4 (10.4–16.4)	9.4 (6.8–12.0)

**Table 8:** Percentage (95% CI) of respondents rating each type of intervention as "harmful" for the person described in the vignette

**Intervention**	**Country**	**Depression Vignette**	**Depression/Suicidal Vignette**	**Early Schizophrenia Vignette**	**Chronic Schizophrenia Vignette**
Physical activity	Australia	0.8 (0.2–2.3)	0.3 (0.1–2.2)	0.4 (0.1–1.3)	0.6 (0.2–1.6)
	Japan	3.6 (2.0–5.2)	4.0 (2.3–5.7)	3.8 (2.1–5.5)	3.6 (2.0–5.2)
Read about problem	Australia	4.1 (2.7–6.2)	5.2 (3.6–7.5)	4.6 (3.1–6.8)	3.6 (2.4–5.4)
	Japan	7.6 (5.3–9.9)	7.8 (5.4–10.2)	8.0 (5.6–10.4)	10.4 (7.7–13.1)
Get out more	Australia	0.4 (0.1–1.7)	0.3 (0.1–1.5)	1.7 (0.9–3.2)	2.2 (1.2–4.1)
	Japan	3.0 (1.5–4.5)	4.8 (2.9–6.7)	7.4 (5.1–9.7)	4.6 (2.8–6.4)
Learn relaxation	Australia	1.5 (0.7–3.2)	0.8 (0.3–2.0)	1.0 (0.5–2.3)	3.6 (2.0–6.1)
	Japan	7.6 (5.3–9.9)	8.2 (5.8–10.6)	16.4 (13.1–19.7)	13.6 (10.6–16.6)
Cut out alcohol	Australia	4.7 (3.1–7.0)	5.3 (3.4–8.0)	3.1 (1.9–5.1)	2.7 (1.6–4.7)
	Japan	17.2 (13.9–20.5)	15.0 (11.9–18.1)	11.4 (8.6–14.2)	12.2 (9.3–15.1)
Psychotherapy	Australia	10.0 (7.5–13.1)	10.6 (8.0–13.9)	5.7 (3.9–8.2)	7.2 (5.2–10.0)
	Japan	7.4 (5.1–9.7)	4.2 (2.4–6.0)	5.2 (3.2–7.2)	2.6 (1.2–4.0)
Hypnosis	Australia	17.0 (13.9–20.7)	20.4 (16.5–24.9)	12.8 (10.1–16.2)	16.7 (13.4–20.5)
	Japan	14.2 (11.1–17.3)	14.0 (10.9–17.1)	17.4 (14.1–20.7)	10.4 (7.7–13.1)
Psychiatric ward	Australia	53.3 (48.4–58.1)	49.2 (44.4–54.1)	38.9 (34.4–43.5)	33.2 (28.9–37.7)
	Japan	43.0 (38.6–47.4)	43.6 (39.2–48.0)	38.0 (33.7–42.3)	24.6 (20.8–28.4)
ECT	Australia	69.4 (64.4–74.0)	65.9 (61.1–70.5)	63.4 (58.5–68.0)	65.4 (60.5–69.9)
	Japan	50.2 (45.8–54.6)	54.4 (50.0–58.8)	50.6 (46.2–55.0)	44.0 (39.6–48.4)
Occasional drink	Australia	15.4 (12.6–18.7)	19.1 (15.6–23.2)	29.8 (25.9–34.1)	25.2 (21.3–29.7)
	Japan	17.4 (14.1–20.7)	20.2 (16.7–23.7)	31.4 (27.3–35.5)	26.8 (22.9–30.7)
Special diet	Australia	7.7 (5.7–10.3)	9.2 (6.9–12.2)	7.7 (5.6–10.6)	7.1 (5.0–9.9)
	Japan	55.2 (50.8–59.6)	55.6 (51.2–60.0)	53.2 (48.8–57.6)	50.4 (46.0–54.8)
Web site	Australia	14.8 (12.0–18.0)	15.3 (12.3–18.8)	12.7 (10.3–15.6)	19.3 (16.2–22.8)
	Japan	8.0 (5.6–10.4)	6.2 (4.1–8.3)	7.2 (4.9–9.5)	9.6 (7.0–12.2)
Expert via email	Australia	14.3 (11.6–17.4)	16.4 (13.1–20.3)	13.8 (11.3–16.8)	17.3 (14.2–20.8)
	Japan	5.0 (3.1–6.9)	5.6 (3.6–7.6)	5.2 (3.2–7.2)	5.8 (3.7–7.9)
Book	Australia	7.7 (5.8–10.1)	9.0 (6.9–11.6)	7.1 (5.1–9.7)	9.4 (7.0–12.4)
	Japan	3.0 (1.5–4.5)	4.4 (2.6–6.2)	4.2 (2.4–6.0)	5.0 (3.1–6.9)
Health educator	Australia	1.4 (0.7–2.8)	2.0 (1.1–3.5)	1.4 (0.7–2.8)	1.6 (0.8–3.2)
	Japan	4.4 (2.6–6.2)	3.8 (2.1–5.5)	5.2 (3.2–7.2)	4.6 (2.8–6.4)

### Beliefs about outcomes

Table 9 gives the data on beliefs about outcomes after receiving professional help and outcomes without professional help. In Australia, the most common belief is that a person receiving professional help would have either full recovery or full recovery with later relapse. In Japan, the public most commonly believed in either full recovery with relapse or partial recovery with relapse.

**Table 9:** Percentage (95% CI) of respondents giving each outcome as likely for the person described in the vignette

**Likely outcome**	**Country**	**Depression Vignette**	**Depression/Suicidal Vignette**	**Early Schizophrenia Vignette**	**Chronic Schizophrenia Vignette**
**With professional help**					
Full recovery	Australia	37.3 (32.8–42.1)	29.6 (25.5–34.1)	24.8 (21.1–29.0)	15.8 (12.4–19.9)
	Japan	7.4 (5.1–9.7)	5.8 (3.7–7.9)	4.4 (2.6–6.2)	2.8 (1.3–4.3)
Full recovery with relapse	Australia	43.6 (39.2–48.1)	48.2 (43.6–52.8)	47.3 (43.0–51.5)	38.9 (34.3–43.7)
	Japan	37.2 (32.9–41.5)	33.8 (29.6–38.0)	34.6 (30.4–38.8)	27.8 (23.9–31.7)
Partial recovery	Australia	9.9 (7.6–12.8)	9.0 (6.9–11.7)	12.8 (10.0–16.2)	19.1 (15.9–22.7)
	Japan	14.8 (11.7–17.9)	15.4 (12.2–18.6)	13.2 (10.2–16.2)	13.6 (10.6–16.6)
Partial recovery with relapse	Australia	5.8 (4.0–8.2)	9.5 (7.1–12.6)	11.9 (9.5–14.9)	21.4 (17.9–25.5)
	Japan	37.4 (33.1–41.7)	40.6 (36.3–44.9)	42.2 (37.9–46.5)	52.8 (48.4–57.2)
No improvement	Australia	0.1 (0.0–0.8)	0.3 (0.1–1.6)	0.3 (0.0–2.3)	0.8 (0.3–2.0)
	Japan	2.4 (1.1–3.7)	1.2 (0.2–2.2)	2.4 (1.1–3.7)	1.6 (0.5–2.7)
Get worse	Australia	0.5 (0.1–1.9)	0.2 (0.0–1.3)	0.2 (0.0–2.4)	0.3 (0.1–1.3)
	Japan	0.0 (0.0–0.0)	0.2 (0.0–0.6)	0.2 (0.0–0.6)	0.0 (0.0–0.0)
Don't know	Australia	2.8 (1.7–4.7)	3.1 (1.9–4.9)	2.8 (1.5–4.9)	3.6 (2.3–5.7)
	Japan	0.8 (0.0–1.6)	3.0 (1.5–4.5)	3.0 (1.5–4.5)	1.4 (0.4–2.4)
**Without professional help**					
Full recovery	Australia	0.6 (0.2–1.9)	0.4 (0.1–1.7)	0.6 (0.2–1.6)	0.1 (0.0–1.2)
	Japan	0.6 (0.0–1.3)	0.8 (0.0–1.6)	0.0 (0.0–0.0)	0.4 (0.0–1.0)
Full recovery with relapse	Australia	2.2 (1.2–4.2)	1.5 (0.8–2.8)	0.7 (0.2–2.3)	0.5 (0.1–1.7)
	Japan	4.2 (2.4–6.0)	2.6 (1.2–4.0)	2.6 (1.2–4.0)	1.2 (0.2–2.2)
Partial recovery	Australia	2.8 (1.6–4.8)	2.5 (1.4–4.6)	0.9 (0.4–2.1)	1.2 (0.5–2.7)
	Japan	3.8 (2.1–5.5)	3.8 (2.1–5.5)	2.6 (1.2–4.0)	1.2 (0.2–2.2)
Partial recovery with relapse	Australia	9.9 (7.4–13.0)	6.7 (4.7–9.3)	3.7 (2.5–5.6)	1.2 (0.5–3.0)
	Japan	12.4 (9.5–15.3)	11.2 (8.4–14.0)	8.6 (6.1–11.1)	4.4 (2.6–6.2)
No improvement	Australia	19.3 (16.2–22.9)	14.2 (11.4–17.5)	14.7 (11.7–18.3)	19.4 (15.9–23.4)
	Japan	29.8 (25.8–33.8)	26.4 (22.5–30.3)	33.6 (29.4–37.8)	39.4 (35.1–43.7)
Get worse	Australia	63.9 (59.4–68.1)	72.0 (67.9–75.9)	78.0 (74.2–81.4)	76.8 (72.2–80.8)
	Japan	47.6 (43.2–52.0)	50.8 (46.4–55.2)	49.0 (44.6–53.4)	53.2 (48.8–57.6)
Don't know	Australia	1.3 (0.6–2.7)	2.7 (1.4–5.0)	1.3 (0.6–2.9)	0.9 (0.3–2.5)
	Japan	1.6 (0.5–2.7)	4.4 (2.6–6.2)	3.6 (2.0–5.2)	0.2 (0.0–0.6)

Where professional help was not received, Australians were most likely to believe the person would get worse. This was also the most common response in Japan, although it was less frequently endorsed than in Australia.

## Discussion

Below we discuss the results from each country separately and then compare the results from the two countries.

### Public beliefs in Australia

The Australian public showed a relatively high level of recognition of depression in the vignettes and this rate was much improved on a similar Australian survey carried out in 1995 [13]. Recognition of the schizophrenia vignettes was not as good, but has also improved since the earlier survey. There was a generally low use of generic lay terms such as "stress", "psychological/mental/emotional problems" and "nervous breakdown". An exception is the generic term "mental illness" which was used by around a quarter of respondents for the early schizophrenia vignette and by around a third for the chronic schizophrenia vignette.

When asked about people who could help, the Australian public showed a high endorsement of GPs and counselors. Psychiatrists were also highly endorsed for the schizophrenia vignettes, more so than in the earlier Australian survey [13]. For medications, only around half endorsed antidepressants for depression, and around a third endorsed antipsychotics for schizophrenia. There were similarly low rates of endorsement of psychotherapy for depression (around half the population) and admission to a psychiatric ward for schizophrenia (around a third). While these endorsement rates are higher than in the 1995 survey [13], they are still low given that these are standard treatments endorsed by most Australian mental health professionals [22,23]. This gap between public and professional beliefs on medication may limit willingness to accept some recommended interventions.

The Australian public sees a range of lifestyle interventions as likely to be helpful, such as increased physical activity, reading about the problem, getting out and about more, and relaxation training. Some of these interventions have supporting evidence for the treatment of depression [24], but not for schizophrenia. In general, the beliefs of the Australian public about treatment are more positive towards lifestyle interventions than towards medical or psychological interventions.

Dealing with the problem alone was seen as likely to be harmful by most Australians, more so than in the earlier survey [13]. A change in such beliefs may help improve the comparatively low rate of help-seeking observed in a 1997 survey of the Australian public [25]. There was also a general belief that seeking professional help would produce a much better outcome for all disorders portrayed in the vignettes, with better outcomes expected for depression than for schizophrenia.

### Public beliefs in Japan

When asked what was wrong with the people portrayed in the vignettes, the Japanese public recognized that there was a mental health problem, but tended to use non-psychiatric labels. Fewer than a fifth used the term "schizophrenia" for the early schizophrenia vignette, but this increased to a third for the chronic schizophrenia vignette. Previous research with Japanese teachers has also shown a low rate of using the term "schizophrenia" in relation to a vignette [26]. This term has very negative connotations in Japan [27], leading psychiatrists to be reluctant to give a diagnosis of schizophrenia to their patients [28]. There have also been proposals to replace the term with a more socially acceptable one [27].

When asked about methods of help, the Japanese public most frequently endorsed counselors, close family and friends. The belief in the helpfulness of counselors has been reported previously in a study of Japanese teachers [26]. Psychiatrists and social workers also received a high level of endorsement for the chronic schizophrenia vignette. Dealing with the problem alone was seen to be harmful by more people than helpful, but still around a fifth of the population saw it as helpful. Previous research has shown that there is considerable stigma on seeking help in Japan and a strong desire for confidentiality, leading some people to seek services far away from their place of residence and to pay cash rather than use health insurance (which would lead to their possible identification) [29]. More people saw psychiatric drugs such as antidepressants and antipsychotics as helpful than harmful, but endorsement of these treatments was not high, consistent with results in other developed countries. Admission to a psychiatric ward was in general not viewed favorably, but was more accepted for chronic schizophrenia. This finding is interesting given the high rate of psychiatric hospitalization in Japan compared to other countries. Dietary changes were also seen very negatively; the reason for this is not clear.

When asked about outcomes, the Japanese public was most likely to believe in partial recovery if the person received professional help, but that the person would get worse if there was no help. There is therefore a general belief that professional help would be beneficial, with better outcomes expected for depression than for schizophrenia.

### Comparison of Australia and Japan

When asked what was wrong with the people portrayed in the vignettes, the Australian public was generally more likely than the Japanese public to use the term "depression" and less likely to use non-psychiatric terms. However, for the schizophrenia vignettes, Australians used the term "schizophrenia" more often for the early schizophrenia vignette than for the chronic vignette, whereas the Japanese applied it more to the chronic vignette. The Japanese public may be reserving psychiatric labels only for the more severe cases of mental disorder.

When asked about sources of help, the most striking difference between the two countries was in attitudes to GPs, who were seen by the Australians as likely to be helpful much more often than by the Japanese. This difference may be related to the nature of the health systems in the two countries. In Australia, GPs are seen as the first point of contact for any health problem and the gateway to other services. There have been efforts in Australia to improve the training of GPs in mental health and to encourage the public to seek help from GPs for mental disorders. In Japan, the role of the GP is an extremely important issue as well, but their interest in psychiatric treatment at the moment is not necessarily great, and it is difficult to say that their ability to diagnose psychiatric patients correctly is sufficient. Currently, calls are growing regarding the importance of re-educating GPs in psychiatric medicine, and in future, if this is realized, GPs should be able to play a suitable role.

Compared to Australians, the Japanese more often endorse the helpfulness of close family and dealing with the problem on one's own, perhaps reflecting a cultural difference in the extent to which mental health issues should be a private matter or perhaps a lack of knowledge. Similarly, the Japanese were much less positive about receiving information from a health educator, but had similar beliefs to Australians about the helpfulness of more private information sources, such as books and the internet.

Australians were much more positive than the Japanese about lifestyle interventions such as diet, physical activity, getting out more, relaxation, and cutting out alcohol. The Australians were also more likely than the Japanese to see psychiatric medications as harmful, particularly tranquillizers and sleeping pills.

The Japanese had a lower rate of endorsing clergy as likely to be helpful, although this was not a highly endorsed form of help in Australia either. In Australia, it is an accepted role of the clergy to support members of their church at times of crisis. In Japan, it has been extremely rare for a conventional clergyman to play any sort of active role in the medical field. However, traditional local shamans, and groups that have been viewed as the so-called "new religions", often offer incantations and prayers for patients, and in many cases mental illness has been described as some sort of "curse", which can be "swept away" by performing suitable rites.

There were also some similarities between the two countries. Both gave a high rate of endorsement of the helpfulness of counselors. In Australia, counselors are not a registered profession and vary greatly in their training. They are seen as providing a supportive role – someone who will listen to problems and give advice. In Japan, counselors are seen to be associated with "mind care", but are not a common profession. The term "counseling" is also used broadly to cover supportive talking relationships that might be provided by people who are not counselors.

Another similarity between the two countries was the predominantly negative view of psychiatric wards and ECT. The negative view of psychiatric wards in both countries is interesting given the much greater use of this intervention in Japan than Australia.

When asked about long-term outcomes, the Japanese were more likely to believe in partial recovery following treatment, while the Australians were more optimistic about full recovery. On the other hand, the Australians were more negative about outcomes without treatment. In both countries, outcomes for depression were seen more positively than outcomes for schizophrenia. A factor that might produce these differences between the two countries is exposure to people with mental disorders. The more hospital-based system in Japan might mean that the public have less contact with people in various stages of recovery.

### Limitations

Both the Australian and Japanese surveys had some methodological limitations. In the Australian survey, the sample was a national one, but there was a considerable amount of non-contact and refusal. In the Japanese survey, the sample was not truly national, but nevertheless covered the country broadly. The representativeness of the sample for the country as a whole is unknown, but is likely to be adequate for investigation of major cross-national differences. Information on refusal was not collected.

Another limitation concerns the problems of making cross-national comparisons between two very different cultures. Because the interview was designed to suit the Australian public, it may not have been optimal for the Japanese. Although we tried to make the survey interviews as close as possible, there will inevitably be subtleties of meaning and cultural factors operating within a structured household survey that could affect the results in unknown ways. For example, there could be differences in the willingness to use various response categories, the acceptability of expressing certain views to an interviewer, or the comparability of interventions or services that may be translated as equivalent. To avoid this limitation as far as possible, we have focused on the broad pattern of responses between countries, rather than small statistically significant differences in percent frequencies.

Some of the interventions listed in the interview are not widely available and respondents would not have had either direct or indirect experience on which to base their beliefs. For example, in Australia, receiving information from a health educator or consulting an expert via email would be rare interventions. Similarly, in Japan, help from priests and naturopaths is rare, while telephone counseling is uncommon but increasing.

The conclusions reached here are limited to the quantitative data collected in community surveys. Future work on cross-cultural comparisons of mental health literacy would benefit from associated qualitative research to document the cultural differences that underpin any quantitative differences found.

## Conclusion

Comparing the two countries, some broad themes are apparent. The Japanese public could be described as more reluctant to use psychiatric labels, particularly with milder disorders, and to be less likely to discuss mental disorders with others outside the family. They generally believe in the benefits of treatment, but are not optimistic about full recovery. By contrast, the Australian public has adopted psychiatric labels, particularly "depression", more than the Japanese. They are more positive about the benefits of seeking professional help, but show a strong preference for lifestyle interventions and tend to be negative about some psychiatric medications. Belief in psychological interventions such as counseling and psychotherapy is similar in the two countries.

In subsequent reports from these surveys we intend to examine differences between the countries in beliefs about causes of mental disorders and in stigmatizing attitudes. These data will allow us to see whether the greater reluctance in Japan to label mental disorders and to expose them outside the family is associated with more negative attitudes or with stigmatizing causal explanations.

## Competing interests

The author(s) declare that they have no competing interests.

## Authors' contributions

AFJ co-designed the Australian survey, analyzed the Australian data, and co-wrote the manuscript.

YN provided overall supervision of the research and provided comments on the manuscript.

HC co-designed the Australian survey and provided comments on the manuscript.

YK provided specific guidance on the Japanese survey, including participation in survey interviewer training, and co-wrote the manuscript.

KMG co-designed the Australian survey and provided comments on the manuscript.

YW was involved in coordination between the Japanese and Australian surveys and co-wrote the manuscript.

## Pre-publication history

The pre-publication history for this paper can be accessed here:


